# Protein Intake and Exercise-Induced Skeletal Muscle Hypertrophy: An Update

**DOI:** 10.3390/nu12072023

**Published:** 2020-07-07

**Authors:** Louise Deldicque

**Affiliations:** Institute of Neuroscience, Université catholique de Louvain, B-1348 Louvain-la-Neuve, Belgium

**Keywords:** protein intake, skeletal muscle mass, net protein balance, hypertrophy, resistance exercise, whey protein, casein

## Abstract

Skeletal muscle mass is critical for sport performance and in many pathological conditions. The combination of protein intake and resistance exercise is the most efficient strategy to promote skeletal muscle hypertrophy and remodeling. However, to be really efficient, certain conditions need to be considered. The amount, type and source of proteins do all matter as well as the timing of ingestion and spreading over the whole day. Optimizing those conditions favor a positive net protein balance, which in the long term, may result in muscle mass accretion. Last but not least, it is also essential to take the nutritional status and the exercise training load into consideration when looking for maintenance or gain of skeletal muscle mass.

Skeletal muscle mass is critical for sport performance in disciplines in which strength is a determinant factor. In certain pathological states, the decrease beyond a minimal amount of skeletal muscle may be vital. It is therefore crucial to develop the most efficient strategies to enhance, in athletes, or to preserve, in patients, skeletal muscle mass. Up to now, combining protein intake and resistance exercise training has revealed the most efficient strategy, taking specific conditions into account. Optimizing resistance exercise training to increase skeletal muscle mass has recently been reviewed by others [1,2,3,4,5]. The purpose of the review by Stokes et al. [6] is to focus on the parameters that influence the efficacy of protein intake in regulating skeletal muscle mass.

Skeletal muscle mass is mainly regulated by the so-called net protein balance, which is defined as the difference between protein synthesis and protein breakdown. On the long term, a positive net protein balance may result in muscle hypertrophy while a negative net protein balance may result in muscle atrophy. In addition to the net protein balance, muscle fiber loss, and the inclusion of satellite cells may contribute to changes in skeletal muscle mass as well. While nutrition in general, and more specifically protein intake, probably contributes to the regulation of satellite cells, only very scarce data have been published [7]. This contrasts with the overwhelming number of studies investigating the effects of nutrition, and more particularly protein intake, in conjunction with resistance exercise and not on muscle protein synthesis and breakdown. Not surprisingly, not all data have converged to consensual conclusions and clear-cut recommendations either for athletes or for patients. The study by Stokes et al. proposes quite an exhaustive and up-to-date overview of the different parameters and conditions influencing the effects of protein intake on the regulation of skeletal muscle mass [6].

The review starts by giving an integrative view of the fate of ingested proteins, underlying the fact that in the post-prandial state, only 10% will actually be used in de novo synthesis in skeletal muscle while 50% are extracted by the splanchnic tissues and the resting 40% are catabolized and contribute to energy production, urea, or neurotransmitter synthesis [8]. However, further research is needed to determine how factors such as protein type [9], age [10], and the gut microbiota [11] may influence those proportions, certainly in the context of resistance exercise.

While animal sources, and particularly whey protein, display a more qualitative amino acid profile than vegetal sources in terms of protein synthesis stimulation [12], there is still some debate about the dose to be ingested after resistance exercise, ranging from 20 to 40 g. Summarizing the data in this field, the authors came to the conclusion that 20 g of high-quality protein (~0.3 g/kg/meal) is sufficient to maximally stimulate muscle protein synthesis after a single meal and when repeated every 3h during the day. This dose may be increased when a whole-body resistance work-out is performed, in somewhat heavier athletes with large muscle mass, and possibly in older individuals [6], though the latter point is still under debate [13].

Another debatable point when looking at the effect of protein intake on muscle protein balance is whether protein should be jointly taken with a source of carbohydrate. The idea is that some amino acid transporters [14] as well as the mammalian target of rapamycin (mTOR) pathway [15], a key regulatory pathway in the stimulation of protein synthesis, are insulin-sensitive. However, insulin levels above ~5 IU/mL, i.e., barely above the fasted state, do not seem to activate muscle protein synthesis [16]. Those results were confirmed by a meta-analysis, concluding that insulin is merely permissive for the stimulation of muscle protein synthesis [17]. Based on this, it is not surprising that studies investigating the potential added value of combining carbohydrate and protein intake post-exercise have failed to observe a superior anabolic response compared to protein ingestion alone [18,19]. Rather, insulin seems to positively regulate protein balance by inhibiting muscle protein breakdown [16]. The question is whether inhibiting protein breakdown would not be detrimental to other key adaptations post-exercise. Knowing that moderate and well-controlled activity of the ubiquitin-proteasome and of autophagy is necessary to remove old and damaged proteins and organelles, blocking those processes could impair protein turnover and cellular adaptations post-exercise [20]. The review by Stokes et al. nicely exposes this issue [6]. The marginal gain, if any, in protein synthesis by carbohydrate-induced insulin production is probably largely counterbalanced by the possible detrimental effects on cellular adaptations post-exercise due to an inhibition of recycling processes such as autophagy. When looking for an increase in muscle mass, as is the case in athletes, the message should be to adopt nutritional strategies to optimize the anabolic response, not to limit protein breakdown. This message differs in patients where the main goal is to limit muscle catabolism [21,22,23]. 

The review ends by reporting the effects of protein intake during energy restriction [6]. Whether voluntary, as is the case in body mass-restricted events and aesthetic disciplines, or involuntary, such as during military operations or in some pathological conditions, a decrease in muscle mass ensues. During energy restriction, protein intake alone is insufficient to maintain lean body mass [24]. While the loss depends on the amplitude of energy restriction, an efficient strategy to maintain lean body mass during this period is to practice resistance exercise and to increase the daily protein intake, up to 3 g protein/kg/day [6]. In addition to protein requirements, the satiety induced by protein intake is an important factor to take into consideration during voluntary energy restriction. From that perspective, whey protein seems to induce a higher degree of satiety compared to casein or soy protein [25]. Altogether, those data have been translated into practical recommendations, distinguishing situations of energy balance from situations of energy deficit [6].

In conclusion, the review by Stokes et al. deserves attention as it dares to question established ideas and it establishes the difference between energy-balanced and -restricted situations, which clearly impacts the fate of the ingested protein.
